# Supplementary material for: Differential ESR1 Promoter Methylation in the Peripheral Blood—Findings from the Women 40+ Healthy Aging Study
Source: Int J Mol Sci. 2020 May 21;21(10):3654. doi: 10.3390/ijms21103654 (PMC7279168; doi:10.3390/ijms21103654)

Supplementary Figure S1. Correlation of *ESR1* CpGI shore methylation between blood and the superior temporal gyrus (STG) [65]. The CpGs presented in the figure are those included as probes in the Illumina system (Illumina 450K probe).

CpG 2 (cg20893956)

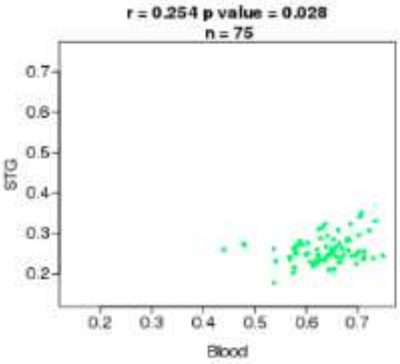

CpG 3 (cg24764793)

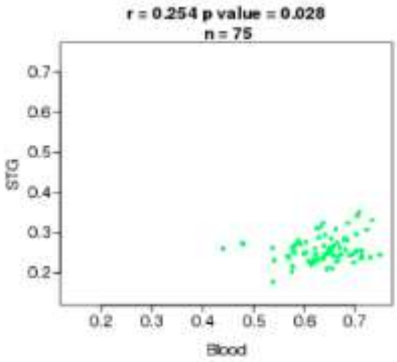

CpG 4 (**cg07746998)**

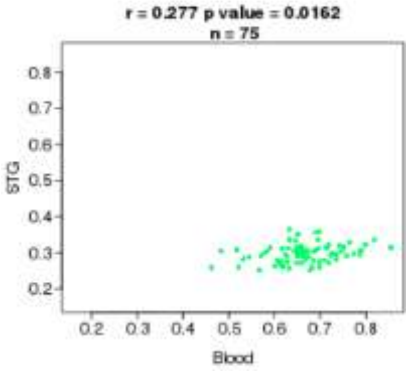

CpG 8 (cg21157690)

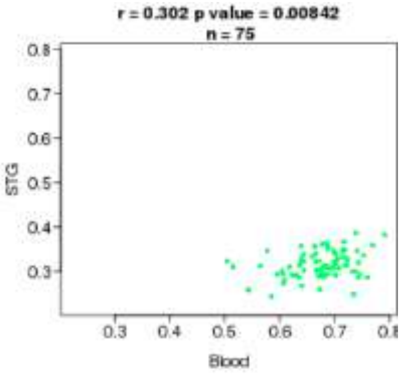

CpG9 (cg17264271)

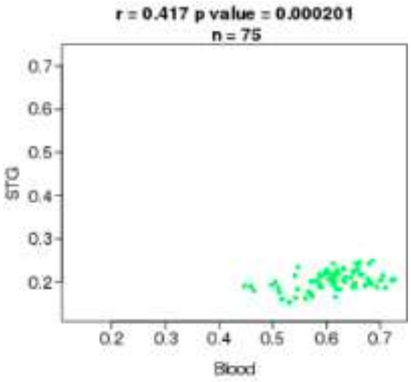

Supplement: Supplementary file 1 [file ijms-21-03654-s001.pdf]
